# *VqMAPKKK38* is essential for stilbene accumulation in grapevine

**DOI:** 10.1038/hortres.2017.58

**Published:** 2017-10-18

**Authors:** Yuntong Jiao, Dan Wang, Lan Wang, Changyue Jiang, Yuejin Wang

**Affiliations:** 1 College of Horticulture, Northwest A & F University, Yangling, Shaanxi 712100, People’s Republic of China; 2 Key Laboratory of Horticultural Plant Biology and Germplasm Innovation in Northwest China, Ministry of Agriculture, Yangling, Shaanxi 712100, People’s Republic of China; 3 State Key Laboratory of Crop Stress Biology in Arid Areas, Northwest A&F University, Yangling, Shaanxi 712100, People’s Republic of China

## Abstract

*Vitis* species, including grapevine, produce a class of secondary metabolites called stilbenes that are important for plant disease resistance and can have positive effects on human health. Mitogen-activated protein kinase (MAPK) signaling cascades not only play key roles in plant defense responses but also contribute to stilbene biosynthesis in grapevine. MAPKKKs function at the upper level of the MAPK network and initiate signaling through this pathway. In this study, a Raf-like MAPKKK gene, *VqMAPKKK38*, was identified and functionally characterized from the Chinese wild grapevine *V. quinquangularis* accession ‘Danfeng-2’. We observed that *VqMAPKKK38* transcript levels were elevated by powdery mildew infection, high salinity conditions and chilling stresses, as well as in response to treatments by the hormones salicylic acid (SA), methyl jasmonate (MeJA), ethylene (Eth) and abscisic acid (ABA). In addition, based on both transient overexpression and gene suppression of *VqMAPKKK38* in grapevine leaves, we found that *VqMAPKKK38* positively regulates stilbene synthase transcription and stilbene accumulation probably by mediating the activation of the transcription factor *MYB14*. In addition, both hydrogen peroxide (H_2_O_2_) and calcium influx activated *VqMAPKKK38* expression and stilbene biosynthesis, which suggests that *VqMAPKKK38* may be involved in the calcium signaling and ROS signaling pathways.

## Introduction

As sessile organisms, plants are constantly exposed to a wide range of biotic and abiotic stresses and have evolved a large number of sophisticated signal transduction mechanisms to both regulate their development and enhance their resistance to these stressors. For example, the mitogen-activated protein kinase (MAPK) cascade is commonly used by eukaryotes to transduce extracellular stimuli into intracellular responses.^1^ The basic components of an MAPK cascade comprise three interconnected kinase modules: MAPKKK/MEKK, MAPKK/MKK and MAPK/MPK.^2^ MAPKKK proteins function in the beginning of the cascade, receiving signals from upstream sensors to initiate the pathway, and activate the MAPKK proteins by phosphorylating the serine/threonine residues in a conserved motif (Ser/Thr-X_3-5_-Ser/Thr, X indicating any amino acid) of the activation loop.^3,4^ The activated MAPKK proteins in turn mediate the activation of downstream MAPK proteins through the phosphorylation of threonine and/or tyrosine residues in the T-X-Y motif.^3,4^ The phosphorylated MAPK proteins then act as regulators of multiple effector proteins in the nucleus or cytoplasm that can be transcription factors, cytoskeletal components and protein kinases.^3,4^ MAPK cascades thus connect upstream signals to downstream targets and participate in the adaptation to a broad range of pathogenic and environmental threats such as bacterial or fungal attack, viral infection, wounding, high salinity, drought, osmotic stress, ultraviolet (UV) irradiation and temperature extremes.^5^

Several three-kinase modules have been functionally characterized in plants. Recent studies in *Arabidopsis thaliana* showed that the MAPKKK17/18-MKK3-MPK1/2/7/14 cascade operates downstream from abscisic acid (ABA)-induced stress signaling^6^ and plays an important role in ABA-modulated leaf senescence.^7^ The AIK1-MKK5-MPK6 module was shown to be activated by ABA and to regulate ABA responses, including root development and stomatal behavior.^8^ Furthermore, two MAPK cascades, MEKK1-MKK1/2-MPK4 and MEKK1-MKK4/5-MPK3/6, have been shown to play a role in flagellin-induced signal transmission,^9^ while the CTR1-MKK9-MPK3/6 cascade is involved in ethylene-regulated signaling.^10^ In addition, in tobacco, the two modules, MAPKKKα-MEK2-SIPK and NPK1-MEK1/NQK1-NTF6/NRK1, contribute to pathogen defense and plant cytokinesis,^11–13^ and tomato (*Solanum lycopersicum*) MAPKKKα-MKK2/MKK4-MPK2/MPK3 is a component of the Pto-mediated effector triggered immunity (ETI) pathway.^14^

Whole-genome sequencing of numerous plant species has generated a valuable resource in the form of an inventory of MAPK families from those species. For instance, there are 20 MAPKs, 10 MAPKKs and at least 80 MAPKKKs in *A. thaliana*, while the grapevine (*Vitis vinifera*) genome contains 14 MAPKs, five MAPKKs and 62 MAPKKKs.^10,15–17^ The MAPKKKs of a given species show enhanced sequence diversity compared to members of the MAPKK or MAPK families.^18^ On the basis of the sequence in the conserved kinase domain, the MAPKKK family in higher plants has been classified into three clades: the MEKK-like subfamily has a conserved signature (G(T/S)Px(W/Y/F)MAPEV); the ZIK subfamily contains a GTPEFMAPE(L/V)Y sequence, and the Raf-like subfamily has a specific GTxx(W/Y)MAPE signature.^18,19^ MEKK1 is the most thoroughly studied MAPKKK in *A. thaliana* and is known to be involved in multiple stress responses, including flagellin signaling,^9^ wounding,^20^ and cold and salt stimuli.^21^ Two well-known Raf-like MAPKKKs, *CTR1* (Constitutive Triple Response 1) and *EDR1* (Enhanced Disease Resistance 1), function as negative regulators of the ethylene ^22^ and pathogen defense responses,^23^ respectively. In cotton (*Gossypium hirsutum*), *GhRaf19* has been shown to enhance the tolerance to chilling stress but to decrease drought and salt stress resistance,^24^ while *GhMKK5* is significantly triggered by salicylic acid (SA) and induces the transcription of pathogenesis-related (PR) genes.^25^ Recently, *A. thaliana AIK1* and *MAPKKK17/18* were identified as key regulators of ABA signal transduction,^7,8^ but relatively little information is available regarding MAPKKKs in grapevine. A genome-wide analysis of MAPK cascades in *V. vinifera* revealed 21 MEKKs, 12 ZIKs and 29 Rafs among the 62 MAPKKKs.^16^ Expression profiles of 45 grapevine MAPKKK genes following exposure to various stress conditions suggested that these candidate MAPKKK genes may participate in responses to powdery mildew, drought, SA, ethylene (Eth) and hydrogen peroxide (H_2_O_2_).^26^ However, a more detailed characterization of individual MAPKKKs is required to better define their biological and physiological roles.

China is a major biodiversity center for *Vitis*,^27^ and Chinese wild grapevines provide valuable gene pools with a number of resistance factors that are thought to be important for pathogen immunity. The Chinese wild grapevine species *V. quinquangularis*, particularly the accession ‘Danfeng-2’, has attracted attention because of its high level of resistance to pathogen infection and its high content of stilbene-type phytoalexins.^28–30^ Stilbenes are secondary metabolites that help promote resistance to a diverse range of pathogens, and they also have pharmacological value.^31–33^ The accumulation of stilbenes can be induced by factors such as pathogen infection,^32^ ozone damage,^34^ wounding,^35^ salt stress^36^ and UV irradiation.^34,37^ Perhaps the most widely studied stilbene, resveratrol, is synthesized by a side branch of the well-characterized phenylalanine/polymalonate pathway where the final step is catalyzed by stilbene synthase (STS).^38^ The R2R3-MYB-type transcription factors MYB14 and MYB15 are thought to regulate the biosynthesis of stilbenes by up-regulating *STS* transcription.^39^ It has also been suggested that MAPK cascades are required for stilbene biosynthesis, and a specific MAPK cascade inhibitor (PD98059) can efficiently suppress the activation of *STS* by flagellin 22 (flg22), a bacterial elicitor harpin,^40^ or by SA.^41^ PD98059 can also block the induction of *MYB14* by flg22 in grapevine cell suspension cultures.^42^

In this study, we sought to identify genes in the MAPK pathway that are involved in the regulation of stilbene accumulation in *V. quinquangularis*. We focused on *VqMAPKKK38*, a Raf-like subfamily member that is responsive to various stressors and examined its role in signal transduction and stilbene biosynthesis. We present functional studies involving transient overexpression and suppression using RNAi in grapevine leaves and propose a mechanism by which *VqMAPKKK38* promotes stilbene biosynthesis, as well as how its involvement in signaling is triggered by calcium and reactive oxygen species (ROS).

## Materials and methods

### Plant materials and stress treatments

Chinese wild *V. quinquangularis* accession Danfeng-2 was cultivated in the grape germplasm resources garden at the Northwest A&F University, Yangling, Shaanxi, China. Samples of young leaves (the second to fourth leaf from the tip), mature leaves (dark-green leaves collected when berries were enlarging), stems (the woody stem), inflorescences (with single flowers in dense groups), young berries (berries were enlarging, 25 days after anthesis) and mature berries (berries were harvest-ripe, 80 days after anthesis) were collected for expression analyses.

Fresh young leaves were collected and subjected to different forms of stress. Powdery mildew (*Erysiphe necator*) inoculation was carried out as described previously,^43^ and inoculated leaves were collected at 0, 12, 24, 48, 72, 96 and 120 h post-inoculation. For abiotic stress treatments, young leaves were either wounded with sterile scissors, sprayed with aqueous 250 mM NaCl, or exposed to low (4 °C) or high temperature (37 °C) for 0, 0.5, 1, 2, 6 or 10 h. Treatment with the signaling molecules was carried out by spraying the young leaves with one of the following solutions: 100 μM SA, 100 μM MeJA, 100 μM Eth, 100 μM ABA, 5 mM CaCl_2_ or 1% H_2_O_2_ (w/v). The leaves were harvested at 0, 0.5, 1, 2, 6 and 10 h post-treatment. The leaves were pretreated with 20 μM GdCl_3_ for 30 min before 5 mM CaCl_2_ was administered to study the roles of Ca^2+^ and ROS. Dimethylthiourea (DMTU), an H_2_O_2_ scavenger, was used to pretreat the leaves for 30 min before 1% H_2_O_2_ was administered. GdCl_3_ or DMTU was also added without a subsequent treatment to assess the effect of the inhibitors on the leaves. Leaves treated with the solvent in which the elicitors were dissolved served as negative controls. Three samples were treated in each treatment, and each treatment was repeated three times.

### Expression analysis

Total RNA was extracted from leaves with the EZNA Total RNA kit II (Omega Bio-Tek) and reverse-transcribed into complementary DNA (cDNA) using Prime Script Reverse Transcriptase (TaKaRa) following the manufacturer’s instructions. Semi-quantitative reverse transcription-PCR was performed with the following parameters: 94 °C for 3 min followed by 30 cycles of 94 °C for 30 s, 58 °C for 30 s and 72 °C for 1 min. The amplified products were separated on a 1% agarose gel and visualized with ethidium bromide. Quantitative real-time PCR (qRT-PCR) was carried out as described previously.^44^ The specific primers used for gene expression analysis are shown in Supplementary Table S1. Gene transcript levels were quantified with normalization to grapevine *GAPDH* (GenBank accession no. GR883080) and *EF1γ* (GenBank accession no. AF176496) as internal standards. Each experiment was carried out with three biological replicates, and each biological sample was analyzed in three technical replicates.

### Cloning and *VqMAPKKK38* sequence analysis

The specific primers used to isolate the full-length *VqMAPKKK38* cDNA (see Supplementary Table S1) were designed according to the homologous sequences from the reference genome of *V. vinifera* cv. ‘Pinot Noir’ clone P40024.^45^ The *VqMAPKKK38* gene is located on the fifth chromosome according to a BLAST search in the Genoscope Genome Browser (http://www.genoscope.cns.fr/blat-server/cgi-bin/vitis/webBlat). DNAMAN software was used to carry out amino-acid sequence alignment analyses of four Raf proteins, including VqMAPKKK38, VviRaf23 (Gene ID: VIT05s0094g01080), AtRaf23 (Gene ID: At2G31800) and BnaRaf23 (GenBank: AHL77715.1). The phylogenetic tree was constructed using MEGA 7 software to analyze the evolutionary relationship between these proteins.

### Plasmid construction and transient expression assays in grapevine

To generate the over-expression construct, the amplified product (open reading frame of *VqMAPKKK38*) was inserted into the pART-CAM-S vector^46^ after digestion with *Sac*I and *Cla*I. To create the silencing construct, a fragment from 545 to 1405 bp of *VqMAPKKK38* was isolated as sense and antisense sequences. Both sense and antisense sequences were cloned into pKANNIBAL,^47^ and then into pART27 ^48^ after restriction digestion by *Not*I. Each sequenced plasmid was transformed separately into *Agrobacterium tumefaciens* strain GV3101 using electroporation and then introduced into 8-week-old *V. quinquangularis* leaves by *Agrobacterium*-mediated transient expression as described previously.^44^

### Stilbene quantification

Stilbene levels in the transgenic leaves were measured as described previously^44^ with minor modifications. The leaf samples were ground in liquid nitrogen, homogenized and extracted with 80% methanol, and then 20 μl of each sample was quantified by high pressure liquid chromatography (Shimadzu Corp, Kyoto, Japan) with a detection wavelength of 306 nm. The mobile phase was 0.5% (v/v) formic acid and acetonitrile (ACN). The mobile phase elution procedure was carried out as follows: 0–8 min, 10–18% ACN; 8–10 min, 18% ACN; 10–15 min, 18–25% ACN; 15–18 min, 25–35% ACN; 18–25 min, 35% ACN; 25–30 min, 35–70% ACN. The sample peaks and standard chemical peaks were calculated using t*rans*-resveratrol and *trans*-piceid (Sigma-Aldrich Inc., http://www.sigmaaldrich.com/) as external standards. We obtained the *cis*-piceid standard by the photoisomerization of *trans*-piceid under UV irradiation. The 50% ethanol solution containing 400 μM of *trans*-piceid was irradiated at 366 nm for 3 h, and the resulting *cis*-piceid standard was stored in total darkness.^37^

## Results

### *VqMAPKKK38* cloning and sequence analysis

**Figure 1:**
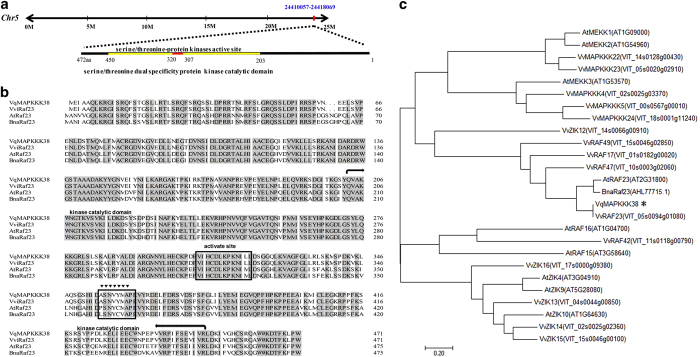
Chromosome location (**a**), sequence alignment analysis (**b**) and phylogenetic analysis (**c**) of *VqMAPKKK38*. The Raf proteins used for the alignment were VviRaf23 (Gene ID: VIT05s0094g01080), AtRaf23 (Gene ID: At2G31800), and BnaRaf23 (GenBank: AHL77715.1). Identical residues are shaded gray. The Raf motif is marked with triangles. The active site (VIHCDLKPKNILL) is boxed, and the kinase catalytic domain is located between the two arrows. The phylogenetic analysis of VqMAPKKK38 and MAPKKK proteins from *Vitis vinifera*, *Arabidopsis thaliana*, *Brassica napus* and *Gossypium hirsutum* was carried out using MEGA 7 software.

The open reading frame (ORF) of *VqMAPKKK38* was amplified by PCR with specific primers using the cDNA derived from leaves of *V. quinquangularis* accession Danfeng-2. The PCR-amplified fragment was 1,419 bp in length and predicted to encode a 472-amino-acid protein with a catalytic kinase domain from residue Gln^203^ to Leu^450^ and a Ser/Thr kinase active site (VIHCDLKPKNILL; Figures 1a and b). A DNA sequence alignment with *VviMAPKKK38* from *V. vinifera* (VviRaf23, ID:VIT05s0094g01080) revealed 100% identity, so the fragment was named *VqMAPKKK38* and considered to be a member of the Raf-like subfamily. A phylogenetic analysis with other MAPKKK proteins and alignment with other Raf-like protein sequences further supported the classification of *VqMAPKKK38* (Figures 1b and c).

### *VqMAPKKK38* expression in different organs and in response to environmental stimuli and hormone treatments

**Figure 2:**
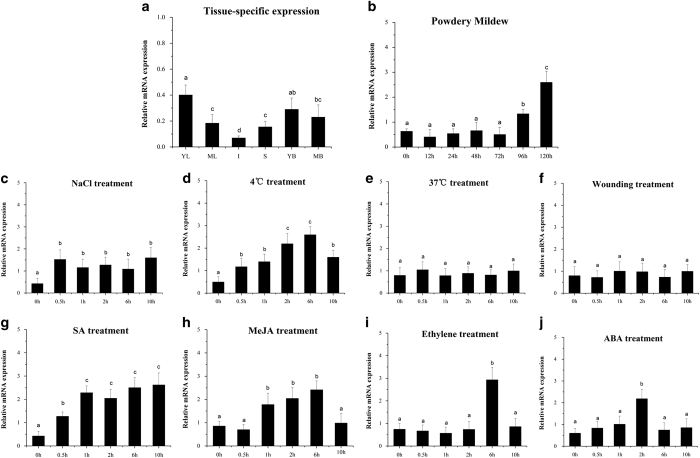
*VqMAPKKK38* expression analysis in various organs and in response to a range of stresses and hormone treatments. (**a**) Expression analysis of *VqMAPKKK38* in different organs determined by qRT-PCR. (**b**) Time course experiment determining *VqMAPKKK38* transcript levels in response to inoculation with powdery mildew, treatment with 250 mM NaCl, chilling, heat and wounding (**c**–**f**), as well as to treatments with 100 μM salicylic acid (SA), methyl jasmonate (MeJA), ethylene and abscisic acid (ABA) (**g**–**j**). Grapevine *GAPDH* and *EF1γ* were used as internal standards. The results are indicated with mean values and s.e. from three biological replicates. Different letters represent significant differences (*P*<0.05) determined by one-way analysis of variance (ANOVA) and *post hoc* comparison test (Student–Newman–Keuls) using SPSS 21.0 for Windows (SPSS Inc., Chicago, IL, USA).

qRT-PCR revealed that *VqMAPKKK38* transcripts were expressed in all of the organs tested, including young leaves, mature leaves, stems, inflorescences, young berries and mature berries. Since the highest levels were observed in young leaves (Figure 2a), these were used for subsequent expression studies.

It has been established that MAPK cascades function in basal immunity ^49^ and are activated in response to various abiotic and biotic stresses.^5^ Therefore, we analyzed the expression profiles of *VqMAPKKK38* during powdery mildew infection, as well as following salt, chilling, heat and wounding treatments. After inoculation with grapevine powdery mildew (*Uncinula necator*), the expression of *VqMAPKKK38* remained at basal levels until 72-hour post inoculation (hpi), and then increased at 96 and 120 hpi. (Figure 2b). Following both salt and chilling treatments, the abundance of the *VqMAPKKK38* transcripts was significantly elevated at 0.5 h, and then remained at high levels throughout the experiment (Figures 2c and d). In contrast, neither the heat or the wounding treatments stimulated *VqMAPKKK38* gene expression for the 10 h duration of the experiments (Figures 2e and f).

Hormones play central roles in plant growth and stress responses, and a number of hormone signals are known to be active via MAPK cascades in plant cells.^50^ We examined *VqMAPKKK38* expression in response to phytohormones and found that SA induced expression within 30 min of treatment, and this persisted over the entire 10 h duration of the experiment (Figure 2g). After MeJA application, the *VqMAPKKK38* transcript levels were strongly up-regulated after 1 h and peaked at 6 h, followed by a sharp decline at 10 h (Figure 2h). Treatment with Eth or ABA led to an upregulation of *VqMAPKKK38* expression at 6 and 2 h, respectively (Figures 2i and j).

### *VqMAPKKK38* promotes stilbene biosynthesis in grapevine

**Figure 3:**
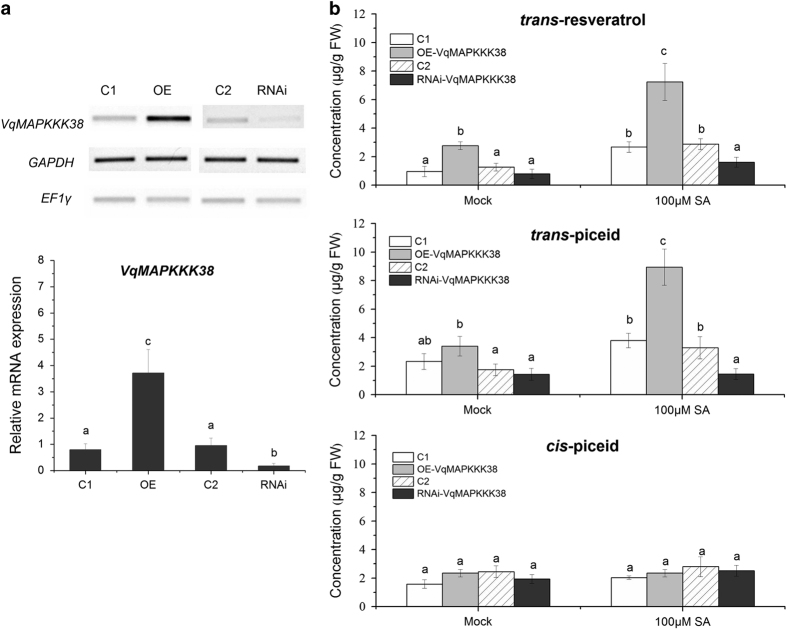
Stilbene accumulation regulated by *VqMAPKKK38*. (**a**) Semi-quantitative RT-PCR (representative agarose gel) and qRT-PCR analysis of *VqMAPKKK38* in transgenic grapevine leaves. C1: grapevine leaves harboring the pART-CAM-S vector without the target gene; C2: grapevine leaves harboring the pART27 vector without the target gene. The housekeeping genes *GAPDH* and *EF1γ* were used as internal standards. (**b**) Amounts of stilbenes in transgenic grapevine leaves after 100 μM salicylic acid (SA) treatment. Mock represents a solvent only treatment. The results represent mean values and s.e. from three independent experiments, and different letters represent significant differences (*P*<0.05) determined by a two-way analysis of variance (ANOVA) and *post hoc* comparison test (Student–Newman–Keuls) using SPSS 21.0 for Windows (SPSS Inc., Chicago, IL, USA).

Our previous studies of grapevine suspension culture cells showed that MAPK signaling is necessary for the activation of *STS* promoters by SA.^41^ As shown in Figure 2, *VqMAPKKK38* expression was strongly induced by SA, so we investigated whether *VqMAPKKK38* plays a role in SA-triggered stilbene biosynthesis. An *Agrobacterium*-mediated transient expression system was used to overexpress (OE) or suppress via RNA interference (RNAi) *VqMAPKKK38* expression in grapevine leaves. pART-CAM-S and pART27 without a target gene served as negative controls. Both semi-quantitative reverse transcription-PCR and qRT-PCR analysis confirmed that *VqMAPKKK38* was successfully overexpressed or silenced (Figure 3a). We measured the stilbene levels in the transformed grapevine leaves before and after 100 μM SA treatment by high-performance liquid chromatography (HPLC). As indicated in Figure 3b, both *trans*-resveratrol and *trans-*piceid levels increased after *VqMAPKKK38* was overexpressed. In addition, after SA treatment, the *VqMAPKKK38* overexpressor accumulated significantly higher levels of these compounds than the control. This suggested that *VqMAPKKK38* promotes stilbene accumulation in grapevine. A transient silencing assay further confirmed this hypothesis, since RNAi-*VqMAPKKK38* leaves exposed to the SA treatment contained much less *trans*-resveratrol and *trans-*piceid than the control leaves. Glucoside *cis*-piceid was also detected in all overexpressing/silenced leaves but showed the same levels as those observed in the controls.

**Figure 4:**
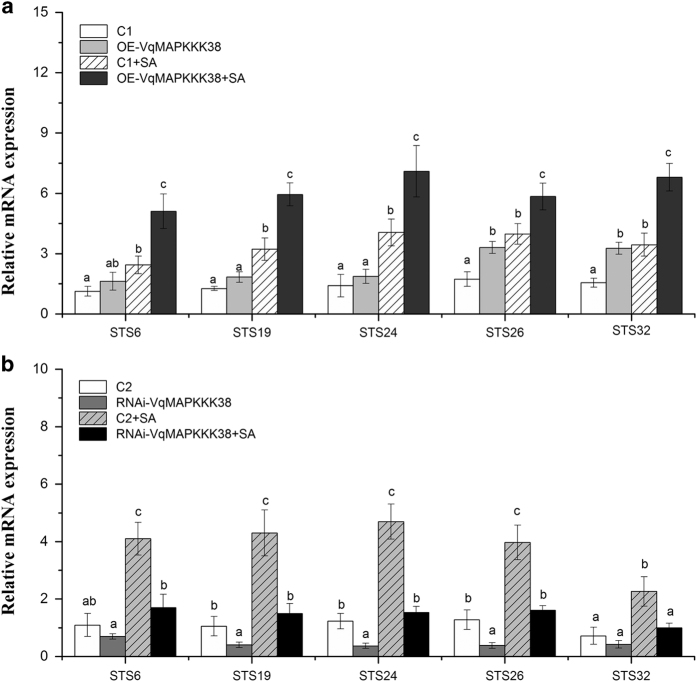
Analysis of *VqSTSs* transcript levels during transient overexpression (**a**) and interference (**b**) of *VqMAPKKK38* in grapevine leaves. Grapevine *GAPDH* and *EF1γ* were used as internal standards for the measurement. The results represent mean values and s.e. from three independent experiments. Mean values with different letters represent significant differences (*P*<0.05) determined by a two-way analysis of variance (ANOVA)and *post hoc* comparison test (Student–Newman–Keuls) using SPSS 21.0 for Windows (SPSS Inc., Chicago, IL, USA).

To investigate whether the upregulation of stilbene biosynthesis caused by *VqMAPKKK38* overexpression was linked to the activation of *STS*, the expression of several *VqSTS* genes was determined. qRT-PCR data showed that the expression of the *VqSTS* genes was significantly induced in OE-*VqMAPKKK38* leaves, especially after SA treatment (Figure 4a). In addition, the induction of the *VqSTS* genes by SA was markedly suppressed in RNAi-*VqMAPKKK38* leaves (Figure 4b). These results are consistent with *VqMAPKKK38* up-regulating the expression of *VqSTSs*.

**Figure 5:**
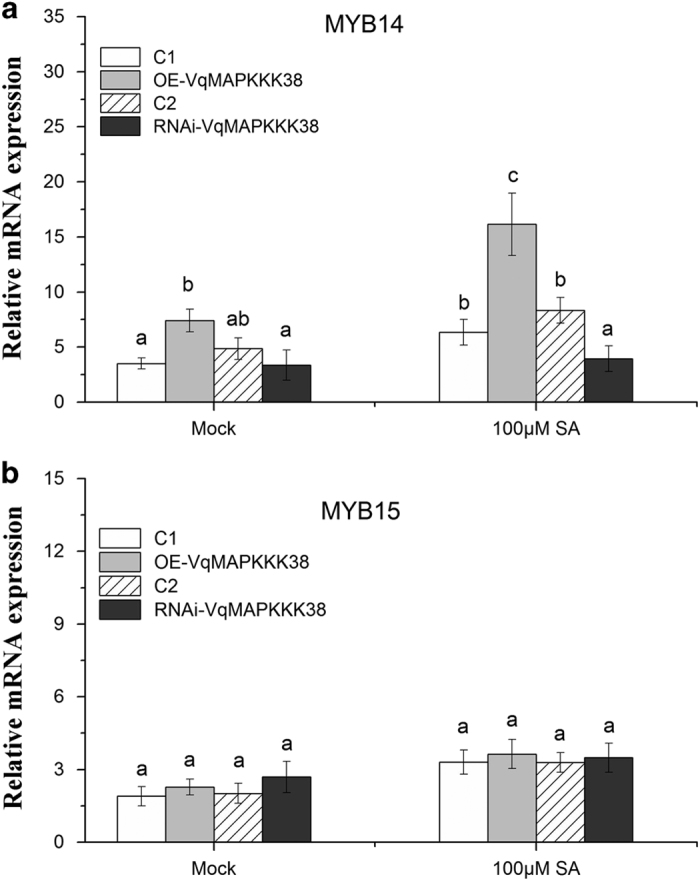
Transcript levels of *MYB14/MYB15* during transient overexpression and interference of *VqMAPKKK38* in grapevine leaves. qRT-PCR data show the transcript abundance of *MYB14* and *MYB15* in transgenic grapevine leaves after 100 μM salicylic acid (SA) treatment. Mock represents transgenic grapevine leaves treated with solvent only. Quantification is relative to *GAPDH* and *EF1γ*. Mean values and s.e. are from three biological replicates. Different letters represent significant difference (*P*<0.05) determined by a two-way analysis of variance (ANOVA) and *post hoc* comparison test (Student–Newman–Keuls) using SPSS 21.0 for Windows (SPSS Inc., Chicago, IL, USA).

It has been shown that the R2R3-MYB-type transcription factors MYB14 and MYB15 are responsible for the regulation of *STS* in grapevine^39^ and that the activation of *MYB14* by flg22 is dependent on MAPK signaling.^42^ To investigate whether the accumulation of stilbenes and the activation of *STS* genes regulated by *VqMAPKKK38* correlated with induction of these transcription factors, we examined their transcript levels in the leaves of the OE- and RNAi-*VqMAPKKK38* lines. In response to SA treatment, the expression of *MYB14* was significantly up-regulated in the OE-*VqMAPKKK38* leaves and downregulated in the RNAi-*VqMAPKKK38* leaves compared with the control (Figure 5a). In contrast, the induction of *MYB15* by SA seemed to be marginal, and we concluded that it was not regulated by *VqMAPKKK38* (Figure 5b).

### *VqMAPKKK38* activation by the calcium ionophore and H_2_O_2_

**Figure 6:**
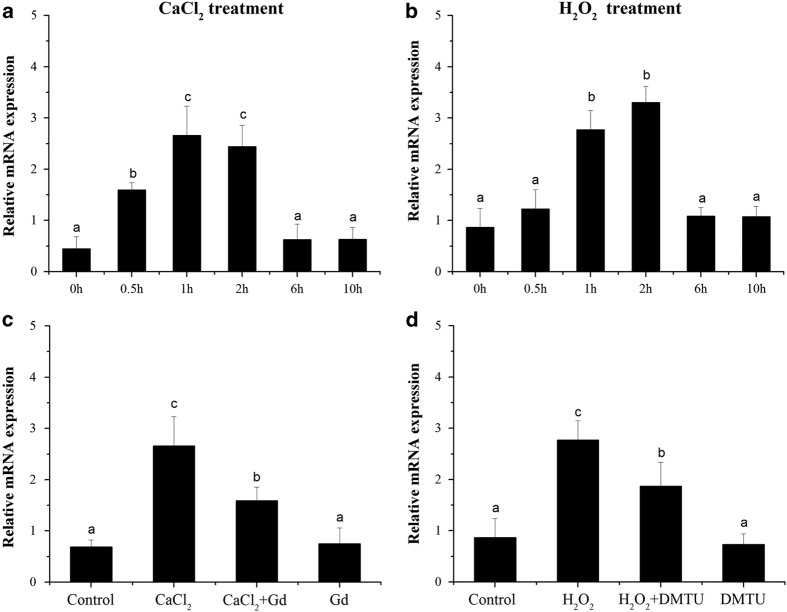
Regulation of *VqMAPKKK38* by calcium influx and hydrogen peroxide (H_2_O_2_). (**a** and **b**) Time course of the expression of *VqMAPKKK38* in response to 5 mM CaCl_2_ and 1% H_2_O_2_ (w/v) treatments, respectively. (**c**) Induction of *VqMAPKKK38* was measured after pretreatment of young *V. quinquangularis* leaves for 30 min with a calcium-influx inhibitor, 20 μM gadolinium chloride (Gd). (**d**) *VqMAPKKK38* expression was measured after pretreatment for 30 min with an H_2_O_2_ scavenger, 5 mM dimethylthiourea (DMTU). *GAPDH* and *EF1γ* were used as internal standards. The results indicate mean values and s.e. from three biological replicates. Different letters represent significant differences (*P*<0.05) determined by a one-way analysis of variance (ANOVA) and *post hoc* comparison test (Student–Newman–Keuls) using SPSS 21.0 for Windows (SPSS Inc., Chicago, IL, USA).

The influx of Ca^2+^, generation of ROS and activation of MAPK cascades are early signaling events associated with immune responses in plants. During unfavorable conditions, these factors are able to trigger and regulate one another,^49,51–54^ leading us to hypothesize that *VqMAPKKK38* expression could be induced by changing the cytoplasmic Ca^2+^ or ROS levels. Young *V. quinquangularis* leaves were treated with 5 mM CaCl_2_ or 1% H_2_O_2_ (w/v) and sampled at 0.5, 1, 2, 6 and 10 h post-treatment. qRT-PCR analysis revealed that *VqMAPKKK38* expression was significantly induced by exposure to either CaCl_2_ or H_2_O_2_ (Figures 6a and b).

To further investigate the role of Ca^2+^ and H_2_O_2_ in *VqMAPKKK38* transcription, we tested the effect of GdCl_3_, an inhibitor of mechanosensitive calcium channels, on the induction of *VqMAPKKK38* by CaCl_2_, as well as the effect of the H_2_O_2_ scavenger dimethylthiourea (DMTU) on the activation of *VqMAPKKK38* by H_2_O_2_. Compared to the solvent controls, the accumulation of *VqMAPKKK38* transcripts significantly increased after adding either CaCl_2_ or H_2_O_2_ for 2 h (Figures 6c and d). When the young leaves were pre-treated with 20 μM GdCl_3_ for 30 min before the CaCl_2_ was administered, the *VqMAPKKK38* expression was higher than that in the solvent control, but significantly lower than in the group treated with CaCl_2_ alone (Figure 6c). The pretreatment of leaves with 5 mM DMTU for 30 min significantly decreased the H_2_O_2_ induction of *VqMAPKKK38* in a manner similar to that of the GdCl_3_ treatment (Figure 6d). Neither GdCl_3_ nor DMTU themselves affected the expression of *VqMAPKKK38*. These results indicated that *VqMAPKKK38* acts downstream of both the calcium and ROS signaling, and so either or both might induce *VqMAPKKK38* expression.

### Stilbene accumulation can be triggered by calcium and H_2_O_2_

**Figure 7:**
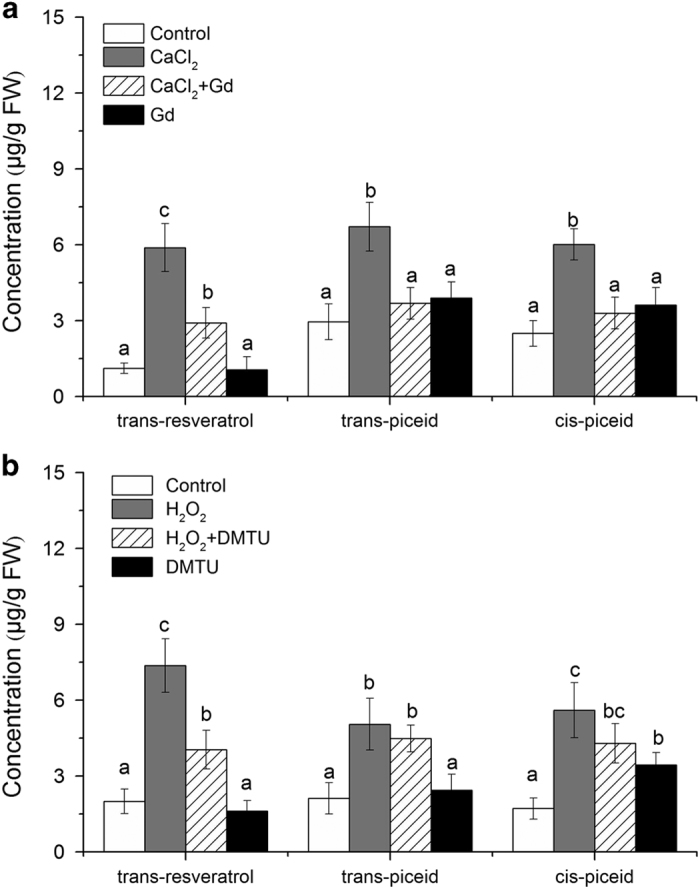
Regulation of stilbene biosynthesis by calcium influx and hydrogen peroxide (H_2_O_2_). Contents of *trans*-resveratrol, *trans*-piceid and *cis*-piceid measured by high pressure liquid chromatography. Mean values and s.e. are from three independent experiments. Different letters represent significant difference (*P*<0.05) determined by a one-way analysis of variance (ANOVA) and *post hoc* comparison test (Student–Newman–Keuls) using SPSS 21.0 for Windows (SPSS Inc., Chicago, IL, USA).

Since *VqMAPKKK38* expression was induced in response to either calcium influx or H_2_O_2_ treatment, we examined the potential correlation between stilbene induction and exogenous calcium and H_2_O_2_. We found that the accumulation of *trans*-resveratrol, *trans*-piceid and *cis*-piceid markedly increased after treatment with either exogenous CaCl_2_ or H_2_O_2_. In addition, the accumulation of the three types of stilbenes induced by CaCl_2_ was significantly limited by the calcium channel inhibitor GdCl_3_, while the H_2_O_2_ scavenger DMTU effectively suppressed the H_2_O_2_-induced accumulation of *trans*-resveratrol (Figures 7a and b). These findings suggest that Ca^2+^ and ROS signaling are involved in stilbene accumulation. qRT-PCR analysis further confirmed that the expression of the *STS* genes was induced in response to calcium influx and ROS signaling. We found that the induction of *VqSTS6*, *VqSTS19*, *VqSTS24* and *VqSTS32* by the calcium ionophore was strongly inhibited by GdCl_3_ (Supplementary Figure 1a). Moreover, the activation of *VqSTS6*, *VqSTS19*, *VqSTS26* and *VqSTS32* by H_2_O_2_ was suppressed when leaves were pretreated with DMTU before the application of H_2_O_2_ (Supplementary Figure 1b).

## Discussion

The resistance to pathogen attack of Chinese wild *Vitis* species such as *V. quinquangularis* accession ‘Danfeng-2’, is correlated with high concentrations of *trans*-resveratrol.^28^ Our previous study of grapevine cell cultures documented the responsiveness of *STS* to SA depended on MAPK signaling.^41^ However, the specific elements of this response pathway were unknown. Recent transcriptome data from four developmental stages of berry material from *V. quinquangularis* accession ‘Danfeng-2’ were analyzed by our colleagues (SRA; SRP067690), and *VqMAPKKK38* was predicted to play a role in the regulation of stilbene accumulation (unpublished). In this study, we investigated the involvement of *VqMAPKKK38* in stilbene biosynthesis and signal transduction.

In grapevine, the biosynthesis of resveratrol is catalyzed by the key enzyme STS that is specifically activated by MYB14.^39^ As previously reported, MAPK signaling can mediate the activation of *STS* transcription.^40,41^ Consistent with this, Duan *et al.*^42^ confirmed that MAPK cascades are essential in the activation of grapevine *MYB14.*^42^ In this study, we show that *VqMAPKKK38* overexpression in grapevine leaves can significantly enhance SA-induced stilbene accumulation, accompanied by the strong induction of *STS* and *MYB14* expression. We also observed that the accumulation of stilbenes was almost abolished in RNAi-*VqMAPKKK38* transgenic leaves, showing that *VqMAPKKK38* is required for stilbene biosynthesis and that a *MAPKKK38*-based cascade is likely to be involved in this process.

The rapid influx of calcium and the generation of ROS are among the earliest cellular responses to biotic and abiotic stresses.^55,56^ The levels of Ca^2+^ and ROS (O_2_^−^, H_2_O_2_, HO· and NO·) are maintained at low levels in plant cells under normal physiological conditions. However, environmental signals can trigger rapid calcium fluxes and increases in the levels of ROS.^57,58^ Either of these responses can activate a number of molecular processes, including MAPK signaling.^54^ In this study, we found that the exposure of young grapevine leaves to either exogenous Ca^2+^ or H_2_O_2_ increased *VqMAPKKK38* expression and that this effect was the most pronounced after 1 and 2 h, respectively. Since the influx of Ca^2+^ and the generation of ROS can directly induce each other,^51,52^ we used GdCl_3_ and DMTU to generate additional evidence for the induction of *VqMAPKKK38* by calcium and H_2_O_2_. Taken together, our results demonstrate that *VqMAPKKK38* functions downstream of both the Ca^2+^ and the ROS signaling pathways and that it responds most rapidly to Ca^2+^-mediated signaling.

The regulation of stilbene accumulation by signaling events has been widely studied in grapevine suspension cell lines. A number of pathogen elicitors can induce stilbene accumulation, including flg22 and harpin, as can hormones such as SA and JA. This inducibility requires an influx of calcium, an oxidative burst and MAPK cascades.^40,41,59,60^ The signaling events are often shared among different induction processes, but different elicitors can generate different types of stilbene output, mainly due to the relative sequence of calcium influx and an apoplastic burst.^40^ Since we found that both calcium and the ROS signaling operate upstream of *VqMAPKKK38*, we examined the effects of calcium and ROS on *STSs* transcription and stilbene biosynthesis. The observation that both the expression of *STS* genes and the accumulation of stilbenes were elevated by Ca^2+^/H_2_O_2_ and could also be inhibited by Ca^2+^/H_2_O_2_ blockers suggested the involvement of Ca^2+^ and ROS signaling in the regulation of stilbene biosynthesis.

It has been shown that exogenous resveratrol can act as a regulator of the hypersensitive reaction accompanied by a stimulation of an oxidative burst in *V. rupestris* suspension cells.^60^ In this study, we found that *VqMAPKKK38* is involved in the ROS signaling pathway, raising the possibility that endogenous stilbenes may in turn regulate the upstream acting *VqMAPKKK38*. We therefore measured the accumulation of *VqMAPKKK38* transcripts in *VqSTS6*-, *VqSTS23*-, or *VqSTS32*-overexpressing transgenic grapevine plants both before and after powdery mildew treatment. However, since we observed no difference in the expression of *VqMAPKKK38* between transgenic and non-transgenic plants (Supplementary Figure S2), we conclude that there is likely no direct feedback regulation between *VqMAPKKK38* and downstream stilbene accumulation in grapevine.

In conclusion, this study provides new insights into the biological roles of a grapevine MAPKKK gene, *VqMAPKKK38*, that has the same coding sequence as *VviMAPKKK38* from *V. vinifera* (ID: VIT05s0094g01080). qRT-PCR analysis revealed that *VviMAPKKK38* expression is strongly induced by *Erysiphe necator*, SA, ethylene and H_2_O_2_,^26^ which is consistent with our report of the expression of *VqMAPKKK38* being induced in response to biotic (*E. necator*) and abiotic (salt, chilling) stresses, as well as defense-related hormone (SA, MeJA, ABA, Eth) treatments. Since the expression profile may be an indicator of gene function, we hypothesize that *VqMAPKKK38* is a stress-inducible gene that is recruited for effective defense against a range of stressors. The evidence from the over- and RNAi-expression experiments with grapevine leaves indicates that *VqMAPKKK38* is involved in a stilbene-type phytoalexin biosynthesis by mediating the transcription of *STS* genes and *MYB14*. Future studies will focus on identification of the *VqMAPKKK38*-mediated expression module and on the use of this gene for molecular breeding of grapevine.
