# Supplementary material for: VqMAPKKK38 is essential for stilbene accumulation in grapevine
Source: Hortic Res. 2017 Oct 18;4:17058–. doi: 10.1038/hortres.2017.58 (PMC5645558; doi:10.1038/hortres.2017.58)
Supplement: Supplementary Figures [file hortres201758-s1.pdf]

Supplementary Figure

VqMAPKKK38 is essential for stilbene accumulation in grapevine

Yuntong Jiao[1,2,3], **Dan Wang**[1,2,3], **Lan Wang**[1,2,3], **Changyue Jiang**[1,2,3], **Yuejin Wang**[1,2,3]*

[1] College of Horticulture, Northwest A & F University, Yangling, Shaanxi, 712100, the People's Republic of China

[2] Key Laboratory of Horticultural Plant Biology and Germplasm Innovation in Northwest China, Ministry of Agriculture, Yangling, Shaanxi 712100, the People's Republic of China

[3] State Key Laboratory of Crop Stress Biology in Arid Areas, Northwest A&F University, Yangling, Shaanxi, 712100, the People's Republic of China

* Corresponding author:

Yuejin Wang

No.3, Taicheng Road, College of Horticulture, Northwest A&F University, Yangling, Shaanxi 712100, the People's Republic of China

Tel: +86-29-87082522

Fax: +86-29-87082522

E-mail: wangyj@nwsuaf.edu.cn

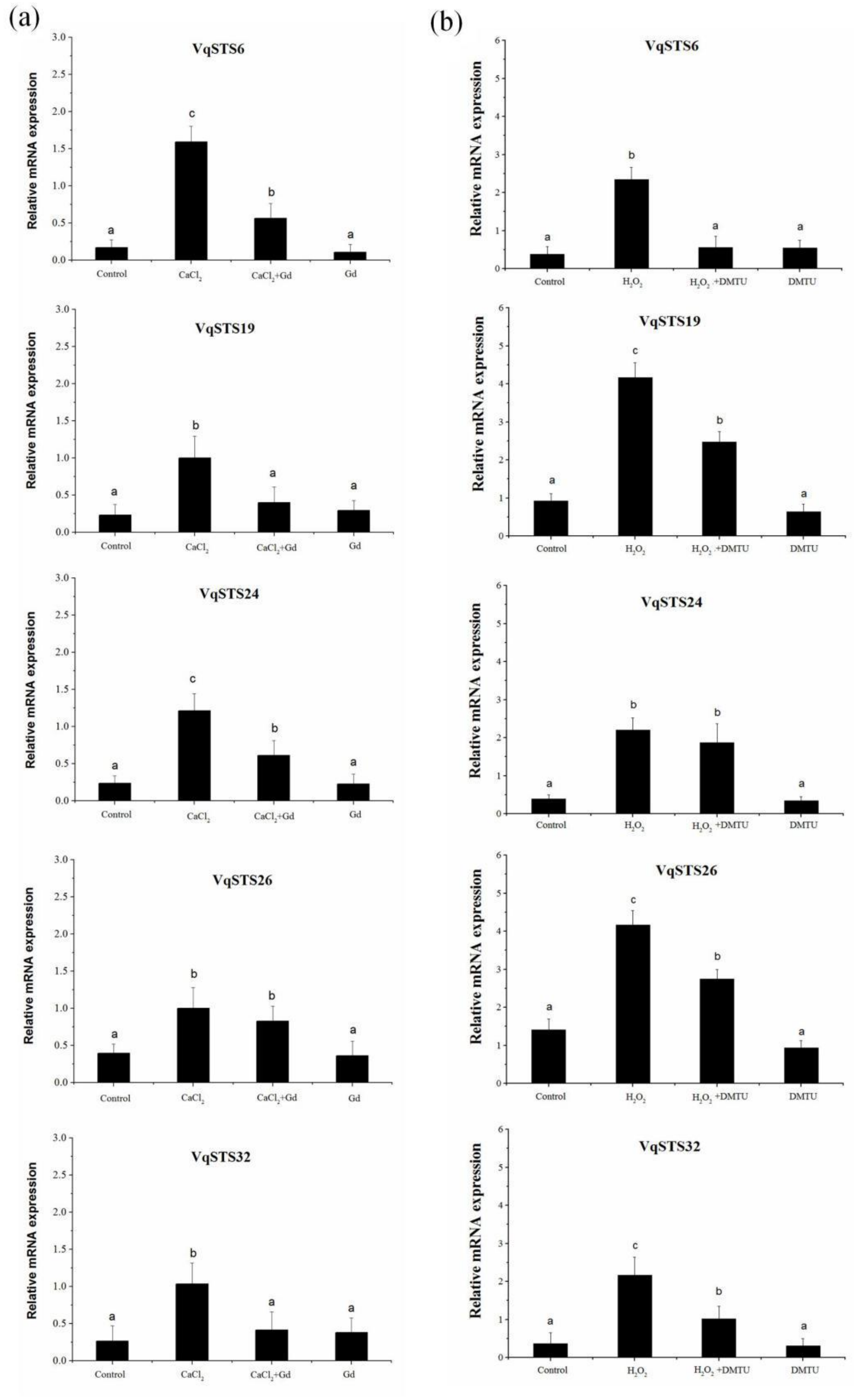

Figure S1. Regulation of *STS* genes by calcium influx and H_2O_2.

Time course of the expression of *VqMAPKKK38* in response to 5 mM $CaCl_2$ and 1% H_2O_2 (w/v). (a) Induction of *STS* gene expression was measured after pretreatment of young *V.] quinquangularis* leaves for 30 min with a calcium-influx inhibitor, 20 μM gadolinium chloride (Gd). (b) Activation of *VqMAPKKK38* was measured after pretreatment for 30 min with an H_2O_2 scavenger, 5 mM dimethylthiourea (DMTU). The *GAPDH* and *EF1γ* genes were used as internal standards. Results show mean values and standard errors from three biological replicates. Different letters represent significant differences ($P<0.05$) based on a one-way ANOVA.

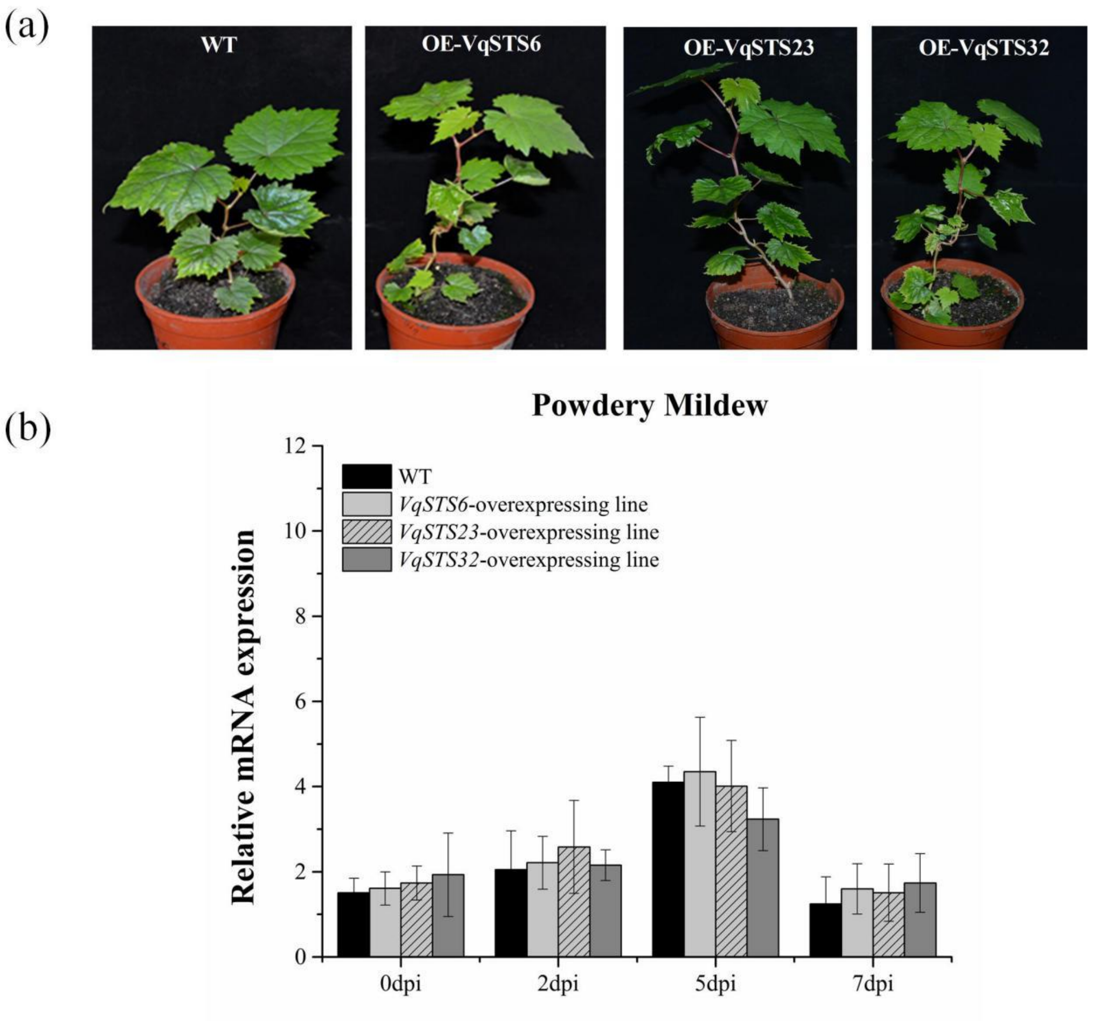

Figure S2. ***VqMAPKKK38*** **expression analysis in STS-overexpressing transgenic grapevine plants in response to powdery mildew inoculation**
