# Supplementary material for: VqMAPKKK38 is essential for stilbene accumulation in grapevine
Source: Hortic Res. 2017 Oct 18;4:17058–. doi: 10.1038/hortres.2017.58 (PMC5645558; doi:10.1038/hortres.2017.58)
Supplement: Supplementary Table S1 [file hortres201758-s2.doc]

**Supplementary Table**

***VqMAPKKK38* is essential for stilbene accumulation in grapevine**

**Yuntong Jiao**^1,2,3^**,** **Dan Wang**^1,2,3^**, Lan Wang**^1,2,3^**, Changyue Jiang**^1,2,3^**, Yuejin Wang**^1,2,3^*****

^1^ College of Horticulture, Northwest A & F University, Yangling, Shaanxi, 712100, the People’s Republic of China

^2^ Key Laboratory of Horticultural Plant Biology and Germplasm Innovation in Northwest China, Ministry of Agriculture, Yangling, Shaanxi 712100, the People’s Republic of China

^3^ State Key Laboratory of Crop Stress Biology in Arid Areas, Northwest A&F University, Yangling, Shaanxi, 712100, the People’s Republic of China

* Corresponding author:

Yuejin Wang

No.3, Taicheng Road, College of Horticulture, Northwest A&F University, Yangling, Shaanxi 712100, the People’s Republic of China

Tel.: +86-29-87082522

Fax: +86-29-87082522

E-mail: [wangyj@nwsuaf.edu.cn](mailto:wangyj@nwsuaf.edu.cn)

**Supplementary Table**

Table S1. Primers used in this study

| **Primer Name** | **Primer sequence 5'-3'** | **References** | **Purpose** |
| --- | --- | --- | --- |
| VqMAPKKK38-F  VqMAPKKK38-R | 5’-ATGGAGATTGCGGCTCAG-3’  5’-TTATTTCCAGGAAGCTTG-3’ | This paper | To clone *VqMAPKKK39* |
| OE-MAPKKK38-F  OE-MAPKKK38-R | 5’-GAGCTCATGGAGATTGCGGCTCAG-3’  5’-ATCGATTTATTTCCAGGAAGCTTG-3’ | This paper | To generate the over-expression construct |
| ds-MAPKKK38-F1  ds-MAPKKK38-R1 | 5’-GGATCCTATGAGCTTAATCCATTAGAG-3’  5’-ATCGATCTTGAAAGTGTCTTTCCA-3’ | This paper | To create the silencing construct |
| ds-MAPKKK38-F2  ds-MAPKKK38-R2 | 5’-CTCGAGTATGAGCTTAATCCATTAGAG-3’  5’-GGTACCCTTGAAAGTGTCTTTCCA-3’ | This paper |
| qRT-MAPKKK-F  qRT-MAPKKK-R | 5’-TGGATAGTGGAGGTCAGTTGAA-3’  5’-TGATGGCTTCTTCTGGAGGTT-3’ | This paper | q-RT-PCR |
| qRT-MYB14-F  qRT-MYB14-R | 5’-GGGGTTGAAGAAAGGTCCAT-3’  5’-GGCCTCAGATAATTCGTCCA-3’ | Duan *et al*., 2015 |
| qRT-MYB15-F  qRT-MYB15-R | 5’-CAAGAATGAACAGATGGAGGAG-3’  5’-TCTGCGACTGCTGGGAAA-3’ | Höll *et al*., 2013 |
| VqSTS6-F  VqSTS6-R | 5’-TTCAATTTCATTACGTATCTAGCATCC-3’  5’-ACAGTGGTCAGGAGTAGCTGTGC-3’ | Shi *et al.*, 2014 |
| VqSTS19-F  VqSTS19-R | 5’-AGAGTGGGGCCAACCAAAGTCTAAG-3’  5’-CCTGCATTATTCTCTGCAAGATCCT-3’ | Shi *et al.*, 2014  q-RT-PCR  Shi *et al*., 2014 |
| VqSTS24-F  VqSTS24-R | 5’-GGTGCGGATTACAAACTTGCTAATC-3’  5’-ACTAAAGAGTCTAAAGCATCCTCGG-3’ | Shi *et al.*, 2014 |
| VqSTS26-F  VqSTS26-R | 5’-CTGTATCTACCAGTCTGATTATGCT-3’  5’-ATTTCTTCAGTCAAGTGAATGTAAC-3’ | Shi *et al.*, 2014 |
| VqSTS32-F  VqSTS32-R | 5’-CAGAGTCACTAAGAGCGAGCAT-3’  5’-ACCGGGCATTTCTACACC-3’ | Shi *et al.*, 2014 |
| VqGAPDH-F  VqGAPDH-R | 5’-TTCTCGTTGAGGGCTATTCC-3’  5’-CCACAGACTTCATCGGTGACA-3’ | This paper | q-RT-PCR |
| VqEF1γ-F  VqEF1γ-R | 5’-GCGGGCAAGAGATACCTCAA-3’  5’-TCAATCTGTCTAGGAAAGGAAG-3’ | This paper |
